# A New Immunological Index for the Elderly: High Proportion of Multiple TCR T Cells Based on scRNA-Seq

**DOI:** 10.14336/AD.2023.0509-1

**Published:** 2024-05-07

**Authors:** Li Jun, Zhu Lanwei, Wang Jiayi, Yao Xinsheng

**Affiliations:** Department of Immunology, Center of Immunomolecular Engineering, Innovation & Practice Base for Graduate Students Education, Zunyi Medical University, Zunyi, China.

**Dear Editor**,

Thymic involution leads to age-related aberrations in T cells which exacerbate the vulnerability to infections, impair the efficacy of vaccinations, and augment autoimmunity and tumor development. These consequences culminate in elevated morbidity and mortality among older adults [1,2]. The T-cell receptors (TCRs) generated during the development of T cells in the thymus play a vital role in the recognition of antigens and the activation of adaptive immune responses. However, studies have found that a few peripheral T cells do not adhere to Burnet’s clonal selection theory, “one lymphocyte—one antigen receptor”, but expressed two TCRs [3-5]. Clonal expansions of dual TCR T cells have also been observed in humans with certain diseases [5, 6]. Currently, the proportion of T cells that bear two distinct TCRs and the potential implications of this phenomenon in the context of age-related thymic involution remain unclear.

Luo et al. [7] described the characteristic features of the immune system landscape in aging using single-cell RNA sequencing, which provided the opportunity to study the dual TCR T cells during the age-related thymic involution. In this study, functional T cells were defined as those T cells that possess at least one functional TCR α sequence and one functional TCR β sequence. As expected, all samples showed the presence of T cells with multiple TCRs. These cells accounted for 9%~16% (Supplementary Table 1) of the total T cells in the peripheral blood. A significant change was observed in the proportion of multiple TCR T cells with age. Multiple TCR T cells were least prevalent in young individuals, and the proportion was significantly lower than that in the elderly population (Fig. 1A). However, the normal T cells with only one TCR exhibited the opposite trend.

The principle of clonal selection suggests that only one allele is used at loci encoding antigen receptors [8]. However, this study detected the presence of multiple α or β chains in a single T cell. The proportion of T cells with “TRB+nTRA” was significant high than T cells with “nTRB+TRA” (n>1) in the old individuals (Fig. 1B), and nearly half of multiple TCR T cells expressed more than one β chain were identified based on the single cell sequencing (Fig. 1C). This exciting discovery poses a challenge to the classical allelic exclusion theory.

The aging process is accompanied by immune function decline, which is characterized by decreased production and function of B and T lymphocytes. Age-related thymic involution not only limits the output of naïve T cells, but also leads to changes in T cell function and subgroup proportions [9]. Additionally, it results in a decreased diversity of the TCR repertoire in the elderly [10]. The present study elucidates a novel form of T cell abnormality that emerges with increasing age, an observed increase in the proportion of T cells expressing multiple TCRs. Whether this phenomenon is beneficial or detrimental to older individuals is still debated. While T cells expressing multiple TCRs have the potential to cause autoimmunity, they also have the potential to expand the TCR repertoire for foreign antigens. In order to investigate whether this phenomenon exists in central immune organs, we also analyzed five single-cell sequencing samples of thymic tissue from the shared database [11]. Despite adequate thymus development in young individuals, multiple TCR T cells were also observed. Age-related thymic involution may be responsible for the high proportion of multiple TCR T cells among the elderly.

**Figure 1.**
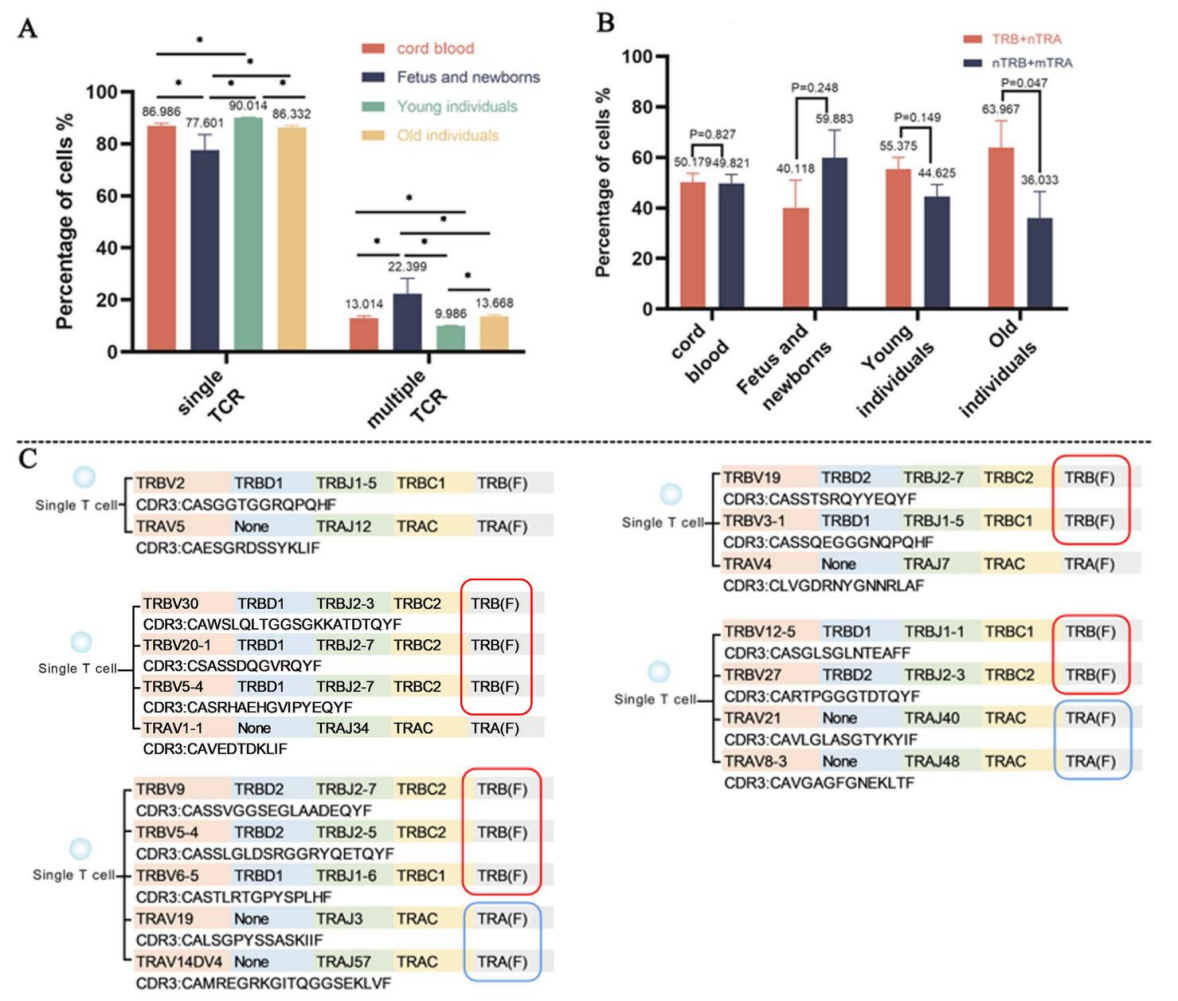
**The multiple patterns of TCR chains based on single-cell immune profiling**. (**A**) statistical analysis of T cells with single TCR and multiple TCRs. (**B**) statistical analysis of multiple TCR T cells that exhibit varied combinations of paired chains. (**C**) examples of multiple different α and/or β chains detected in single T cells. TRB: TCR β chain; TRA: TCR α chain; F: functional. *: *P*<0.05.

Currently, the origin and potential immunological functions of multiple TCR T cells are not well understood. The expression of multiple TCRs on single T cells may be associated with peripheral T cell lifespan, transplant rejection, and the onset and progression of autoimmune diseases. Additional research is needed to comprehend the potential immunological roles of multiple TCR T cells and to clarify the mechanisms of V(D)J allelic exclusion.

## Supplementary Materials

The Supplementary data can be found online at: www.aginganddisease.org/EN/10.14336/AD.2023.0509.


