# Supplementary material for: A New Immunological Index for the Elderly: High Proportion of Multiple TCR T Cells Based on scRNA-Seq
Source: Aging Dis. 2024 May 7;15(3):948–50. doi: 10.14336/AD.2023.0509-1 (PMC11081166; doi:10.14336/AD.2023.0509-1)
Supplement: Supplementary file 1 [file AD-15-3-948-s.pdf]

SUPPLEMENTARY DATA

A New Immunological Index for the Elderly: High Proportion of Multiple TCR T Cells Based on scRNA-Seq

Li Jun, Zhu Lanwei, Wang Jiayi, Yao Xinsheng

© 2023. Jun L et al. Published online at http://www.aginganddisease.org/EN/10.14336/AD.2023.0509-1

SUPPLEMENTARY DATA

Supplemental Table 1. The basic information of T cells with single or multiple TCRs.

Samples	Accession number	Total T cells	T cells (%)			
			One TCR	Multi-TCR	TRB+ nTRA	nTRB+ mTRA
Cord blood	GSM4750320	1837	88.459	11.541	5.988	5.553
	GSM4750321	2741	86.976	13.024	7.187	5.837
	GSM4750322	3067	85.523	14.477	6.293	8.184
Young blood	GSM4750317	2437	90.562	9.438	5.252	4.185
	GSM4750318	2262	89.920	10.080	4.775	5.305
	GSM4750319	2941	89.629	10.371	5.202	5.168
Elderly blood	GSM4750323	2074	88.042	11.958	9.402	2.555
	GSM4750325	3431	87.205	12.795	7.112	5.683
	GSM5684309	3437	85.569	14.431	10.911	3.521
	GSM5684310	4122	84.619	15.381	4.051	11.329
	GSM5684311	4182	86.227	13.773	11.526	2.248

$n \geq 2$, $m \geq 1$. We appreciate the single-cell V(D)J sequencing data shared by Luo et al. and Park et al.

© 2023. Jun L et al. Published online at http://www.aginganddisease.org/EN/10.14336/AD.2023.0509-1
